# Impact of Suppressor of Mothers Against Decapentaplegic (SMAD) 7 Gene Single Nucleotide Polymorphisms on Colorectal Cancer Risk and Prognosis

**DOI:** 10.7759/cureus.70081

**Published:** 2024-09-24

**Authors:** Maurício Peixoto, Marta Viana-Pereira, Maria Júlia Amorim, Ana D Marques, Diana Freitas, Joana Cunha, Filipa Pereira, Rui Reis, Elisabete Couto

**Affiliations:** 1 Medical Oncology, Unidade Local de Saúde de Braga, Braga, PRT; 2 Oncology, School of Medicine and the Life and Health Sciences Research Institute, University of Minho, Braga, PRT; 3 Molecular Oncology Research Center, Barretos Cancer Hospital, Barretos, BRA

**Keywords:** colon cancer, colorectal cancer, prognosis, rectal cancer, risk, smad7

## Abstract

Colorectal cancer (CRC) is a prevalent diagnosis worldwide with significant associated mortality. Single nucleotide polymorphic (SNP) variants have been identified as being associated with CRC risk. Although SMAD7 SNPs have been associated with the risk of developing CRC, their prognostic effect is still unclear.

We carried out a case-control study to establish an association between genotypes of the suppressor of mothers against decapentaplegic (SMAD) 7 SNP rs4464148, rs4939827, and rs12953717 and CRC risk. Furthermore, we retrospectively assessed whether these SNPs had prognostic implications in CRC patients by evaluating survival with Kaplan-Meier curves and Cox regression.

Only the CT genotype of the rs4939827 variant showed an association with CRC risk, and no genotype (CC, CT, or TT) of any of the three SNPs was shown to have prognostic implications in overall survival.

Our study failed to show an association between certain SNP genotypes and the risk of CRC, which has already been well documented in two meta-analyses. Furthermore, it showed no prognostic relevance for these SNPs. More studies are needed to understand whether there are population variations or haplotype effects that could disturb the evaluation of these results.

## Introduction

According to data from GLOBOCAN 2022, colorectal cancer (CRC) is the third most common cancer, preceded only by breast and lung cancer, and accounts for 9.3% of cancer mortality worldwide, second only to lung cancer [1]. In Portugal, CRC is the most frequent cancer, totaling 15.2% of new cancers diagnosed in 2022, and is responsible for 14.2% of deaths, only behind lung cancer [2].

Most cases of CRC are sporadic and are associated with various risk factors, such as obesity, a sedentary lifestyle, red meat consumption, alcohol consumption, and smoking [3]. Besides the above-mentioned etiological factors, the genetic background of each individual, particularly their single nucleotide polymorphic variants (SNPs), plays an important role in CRC risk [4].

Over the last decades, genome-wide association studies (GWAS) have identified more than 100 independent common genetic variants associated with CRC risk [4]. Some of these variants are located in the intronic region of the suppressor of mothers against decapentaplegic (SMAD) 7 gene, namely rs4464148, rs4939827, and rs12953717, and are associated with adenomas and colorectal carcinoma risk [5,6]. The protein encoded by *SMAD7* acts negatively on the TGF-β signaling pathway, a complex signaling pathway that appears to control the inflammatory state of the intestinal mucosa [7]. In addition, through a TGF-β-independent pathway, it also appears to be associated with STAT3 expression and cell pluripotency [8,9].

Previously, two meta-analyses concluded that the rs4464148 (T>C) and rs12953717 (C>T) variants, as well as the T allele of the rs4939827 variant, are associated with a higher risk of CRC [10,11]. Despite these results, no studies have validated their impact on the Portuguese population, which is poorly represented in GWAS studies. Moreover, their role in the prognosis of the disease has not yet been well studied.

The aim of this study was to verify the association of *SMAD7* SNPs with the risk of CRC and to assess whether they have a prognostic effect on the overall survival of CRC patients.

## Materials and methods

Population

This case-control study evaluated 399 participants, comprising 202 patients diagnosed with CRC between 1999 and 2010 and 197 healthy controls. All patients signed an informed consent form and had peripheral blood collected for genomic characterization of *SMAD7*. The patients were all treated at Braga Hospital, Portugal, and had to have colon or rectal adenocarcinoma histology, staged with colonoscopy, thoraco-abdominopelvic CT, and pelvic MRI (replacing pelvic CT in the case of rectal tumors). Treatment followed the guidelines of the European Society for Medical Oncology (ESMO) available when the therapeutic decision was made. Treatment schemes included 5-fluorouracil, capecitabine, irinotecan, oxaliplatin, bevacizumab, cetuximab, panitumumab, and trifluridine-tipiracil.

Patients' clinical data included histologic type (defined by the WHO [12]) and degree of differentiation, cancer stage at the time of diagnosis (following the American Joint Committee on Cancer (AJCC) staging manual [13]), the follow-up time (number of months between diagnosis and last observation or death of the patient), and the current survival status (alive with no evidence of disease; alive with evidence of disease; dead without evidence of disease; dead with evidence of disease; lost to follow-up, if the last observation had occurred more than 12 months before).

The 197 controls were cancer-free random blood donors, selected by adjusting for the gender and age of the patients. All patients in both groups were representative of the population in the north of Portugal, mostly Caucasians.

The study was previously approved by local ethical authorities, and all participants signed an informed consent form to have the *SMAD7* gene genotyped. Data anonymity and confidentiality were maintained throughout data collection and processing.

*SMAD7* genotyping

*SMAD7* genotyping was performed on DNA obtained from nucleated peripheral blood cells using the Citogene® commercial kit according to the manufacturer's recommendations. After extraction, the DNA was analyzed by agarose gel electrophoresis, quantified by nanodrop, and stored at minus 70°C until use.

The *SMAD7* gene is located at locus 18q21.1 and the three SNPs analyzed were rs4464148, rs4939827, and rs12953717. Genotyping was carried out using the Sequenom MassARRAY iPLEX Gold platform (Sequenom, San Diego, California) at the Instituto Gulbenkian de Ciência, in Lisbon. The primers were obtained using MassARRAY Assay Design 3.1 software (Sequenom, San Diego, California). The quality of the genotyping was assessed by duplicating the analysis of 10% of the samples.

Statistical analysis

The statistical analysis of the results was carried out using the SPSS program, version 26.0. The Χ2 test was used to verify that the allelic distribution in the control group was in Hardy-Weinberg equilibrium (HWE). The OR and 95% CIs for the effect of SNPs on the risk of CRC were calculated using multivariate logistic analysis (adjusted for gender and age).

Patients’ overall survival was defined by the period between the diagnosis and the time of death (if known) or the latest observation and was assessed using Kaplan-Meier curves.

The association between SNPs and overall survival was analyzed using the Cox regression method, through the HR and respective 95% CI.

## Results

Characterization of the population

The epidemiological and clinicopathological features of patients and controls are summarized in Table 1. The median age of the CRC patients at diagnosis was 66 years old, while that of the controls was 50. Of the 202 patients, 63.4% were men, similar to 65.5% of the control population. There was a family history of CRC in 18.3% of the patients. The vast majority had histology compatible with adenocarcinoma without specificity, with the remaining 7.4% being mucinous. Just over a third had adenocarcinoma of rectal origin. Most patients had stage III disease (35.6%) (Table 1).

**Table 1:** Characteristics of the study population.

	Cases n=202	Controls n=197
Age		
Median	66	50
Minimum	34	24
Maximum	92	85
Sex		
Male	128 (63.4%)	129 (65.5%)
Female	74 (36.6%)	68 (34.5%)
CRC Family History		
Yes	37 (18.3%)	
No	107 (53%)	
Unknown	58 (28.7%)	
Location		
Colon	124 (61.4%)	
Rectum	78 (38.6%)	
Histologic Type		
Adenocarcinoma NOS	187 (92.6%)	
Mucinous Adenocarcinoma	15 (7.4%)	
Stage		
I	26 (12.9%)	
II	52 (25.7%)	
III	72 (35.6%)	
IV	52 (25.7%)	
Follow-up Time (months)		
Median	77	
Minimum	0	
Maximum	202	

Association with CRC risk and prognosis

The SMAD7 genotypes are shown in Table 2.

**Table 2:** Genotype distribution in each group for each SNP. SNP: Single nucleotide polymorphism.

SNP	Genotype	Controls (n(%))	Cases (n(%))
rs4464148	TT	81(41.1%)	82 (40.6%)
	CT	93 (47.2%)	96 (47.5%)
	CC	23 (11.7%)	24 (11.9%)
rs4939827	TT	56 (28.4%)	63 (31.2%)
	CT	92 (46.7%)	103 (51.0%)
	CC	49 (24.9%)	36 (17.8%)
rs12953717	CC	55 (27.9%)	49 (24.3%)
	CT	100 (50.8%)	98 (48.5%)
	TT	42 (21.3%)	55 (27.2%)

For the rs4464148 SNP, the CT genotype was the most frequent, present in 47.2% of the controls and 47.5% of the cases. Similarly, for the rs4939827 and rs12953717 SNPs, the CT genotype was the most frequent, with 46.7% of controls and 51.0% of cases and 50.8% of controls and 48.5% of cases, respectively. The allelic frequency for each SNP is shown in Table 3.

**Table 3:** Allelic frequency for each SNP. SNP: Single nucleotide polymorphism.

SNP	Allelic Frequency	Controls	Cases
rs4464148	T	0.647	0.644
	C	0.353	0.356
rs4939827	T	0.518	0.567
	C	0.482	0.433
rs12953717	C	0.533	0.485
	T	0.467	0.515

An association was found between the risk of CRC and age (OR 1.116; 95% CI: 1.091-1.142; p < 0.001) and with the CT genotype of the rs4939827 variant (OR 2.376; 95% CI: 1.033-5.465; p = 0.042). There was also a tendency for a positive correlation with the TT genotype of the rs12953717 variant (OR 3.874; 95% CI: 0.990-15.153; p = 0.052), as shown in Table 4.

**Table 4:** Results of multivariate logistic regression analyzing the association between SNP genotypes, age, and sex with CRC risk. SNP: Single nucleotide polymorphism; CRC: Colorectal cancer.

SNP		Controls (n(%))	Cases (n(%))	OR (95% CI)	P-value
rs4464148	TT	81 (41.1%)	82 (40.6%)	-	
	CT	93 (47.2%)	96 (47.5%)	1.157 (0.623-2.148)	0.644
	CC	23 (11.7%)	24 (11.9%)	0.962 (0.340-2.720)	0.942
rs4939827	TT	56 (28.4%)	63 (31.2%)	-	
	CT	92 (46.7%)	103 (51.0%)	2.376 (1.033-5.465)	0.042
	CC	49 (24.9%)	36 (17.8%)	2.436 (0.699-8.490)	0.162
rs12953717	CC	55 (27.9%)	49 (24.3%)	-	
	CT	100 (50.8%)	98 (48.5%)	1.097 (0.416-2.892)	0.851
	TT	42 (21.3%)	55 (27.2%)	3.874 (0.990-15.153)	0.052
Age		197	202	1.116 (1.091-1.142)	<0.001
Sex	Male	129 (65.5%)	128 (63.4%)	-	
	Female	68 (34.5%)	74 (36.6%)	1.083 (0.658-1.782)	0.754

Using allelic frequency, and controlling for age and sex, an association was found between CRC risk and the rs12953717’s T allele (OR 3.960; 95% CI: 1.027-15.262). A negative tendency was also found with the T allele of the rs4939827 SNP and CRC risk (Table 5).

**Table 5:** Multivariate logistic regression results for rs4464148 C allele, rs4939827 T allele, and rs12953717 T allele in CRC, controlled for age and sex. CRC: Colorectal Cancer; SNP: Single Nucleotide Polymorphism.

SNP	OR (CI 95%)	P
rs4464148 C allele	1.077 (0.410-2.829)	0.881
rs4939827 T allele	0.353 (0.104-1.197)	0.095
rs12953717 T allele	3.960 (1.027-15.262)	0.046

**Figure 1:**
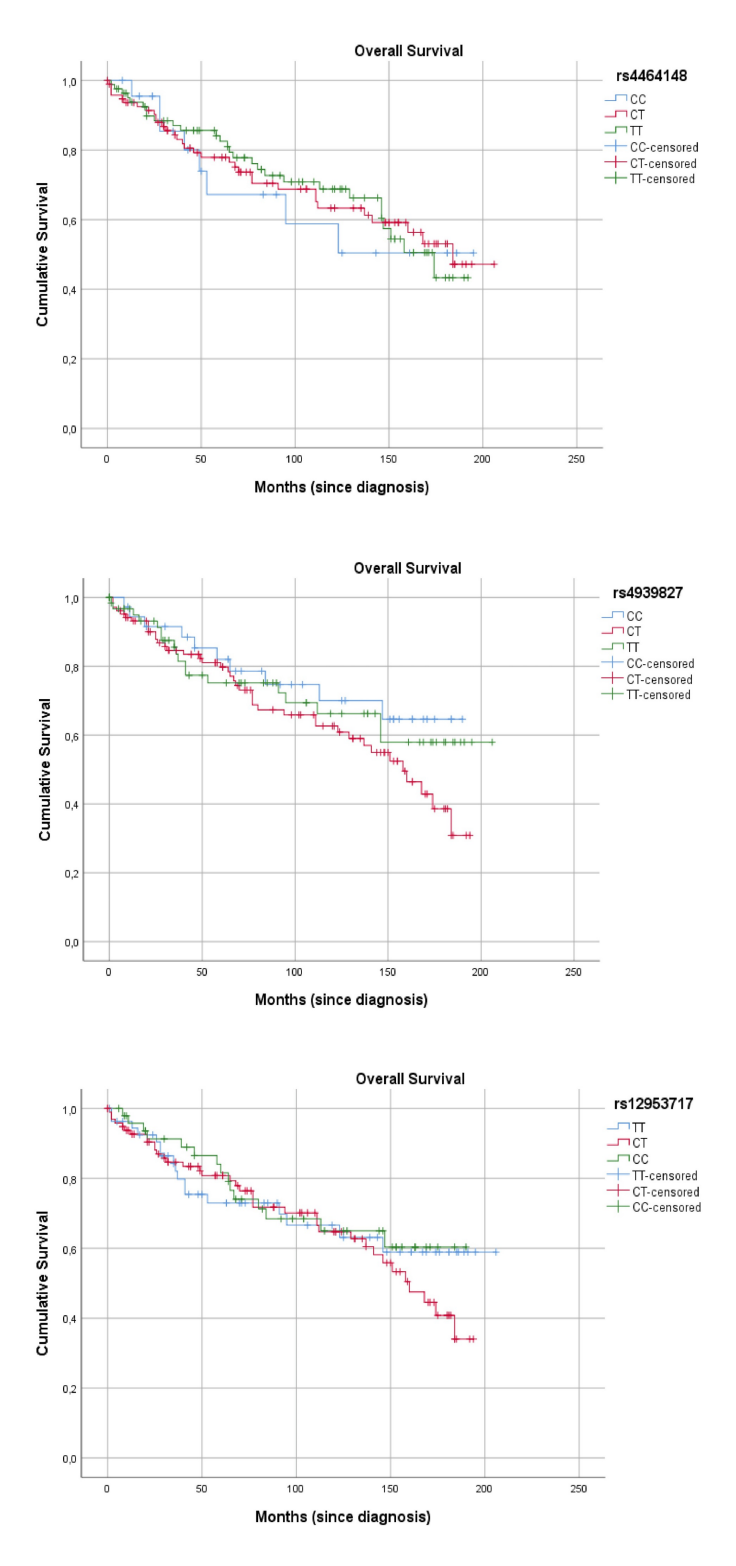
Overall survival curves per SNP genotype. SNP: Single Nucleotide Polymorphism.

Regarding the prognostic effect, the survival curves by genotype of each SNP and by stage can be seen in Figure 1.

There were no statistically significant differences in overall survival from diagnosis between the genotypes of each SNP, even using dominant or recessive models (Table 6), and/or controlling for patients with metastasis at diagnosis (stage IV) (data not shown).

**Table 6:** Cox regression test results for SNP genotypes, including dominant and recessive models. SNP: Single Nucleotide Polymorphism.

SNP	Cox test	HR (95% CI)
rs4464148	CC vs. CT	0.884 (0.408-1.915)
	CC vs. TT	0.867 (0.394-1.904)
	CC vs. CT+TT	0.876 (0.419-1.832)
	CC+CT vs. TT	0.958 (0.592-1.552)
rs4939827	CC vs. CT	1.644 (0.823-3.284)
	CC vs. TT	1.210 (0.558-2.622)
	CC vs. CT+TT	1.482 (0.758-2.898)
	CC+CT vs. TT	0.829 (0.484-1.420)
rs12953717	TT vs. CT	1.231 (0.693-2.188)
	TT vs. CC	0.941 (0.469-1.888)
	TT vs. CT+CC	1.131 (0.654-1958)
	CT+TT vs. CC	0.820 (0.462-1.454)

## Discussion

The *SMAD7* polymorphisms have been associated with CRC risk. In the present case-control study of a Portuguese population, we analyzed 202 cases and 197 controls from a single institution and evaluated the impact of rs4464148, rs4939827, and rs12953717 on cancer risk and prognosis.

Two meta-analyses [10,11] have shown the association between the risk of CRC and each of the SNPs evaluated in this study. Huang Y et al. assessed the existing evidence for the association between these three SNPs and the risk of CRC. The SNP rs4464148 (T>C) was associated with increased risk, mainly in a recessive model in the Caucasian and Asian populations. The rs4939827 (C>T) variant was associated with increased risk in both dominant and recessive models in Caucasians. The rs12953717 (C>T) variant showed an association with increased risk in both recessive and dominant models in Caucasians and Asians. More recently, Xiao Q et al. also assessed the association between these three SNPs and the risk of CRC. The authors showed that the rs4939827 (T>C) SNP was associated with a reduced risk of CRC in Caucasians, and both the rs4464148 (T>C) and the rs12953717 (C>T) variants were associated with an increased risk of CRC in Caucasians [11].

In our study, we validated that the CT genotype of the rs4939827 variant was associated with an increased risk of CRC, but this was not the case for the CC genotype. Using allelic frequency, we observed a positive tendency for decreased CRC risk with the T allele, although statistical significance was not reached. This is contrary to what was shown in the cited meta-analysis.

We also found that the rs12953717 T allele was associated with increased CRC risk, in agreement with what was previously reported [11], even though this association was not found with either the CT or TT genotypes. No other risk associations were observed.

Regarding the prognostic effect, no differences were found between the genotypes of each variant in overall survival, as observed from the Kaplan-Meier curves and the Cox regression analysis. Distinct results were reported by Hu Y et al. in patients with rectal cancer who had undergone surgery [14]. The authors found that the T allele of the rs12953717 variant was associated with shorter disease-free survival (DFS), while the T allele of rs4939827 showed a trend in this direction, but without statistical significance. The C allele of SNP rs4464148 also showed an association with shorter DFS, with an additive effect (worse in homozygosity compared to heterozygosity), and this effect was also seen in shorter overall survival.

The biological effects of these intronic *SMAD7* variants are not clear. The work by Hu Y et al. showed no association between the genotypes and different levels of *SMAD7* expression, suggesting that other linkage effects could be present [14]. Fortini BK et al. [15] demonstrated that four variants in linkage disequilibrium (LD) (r2 = 1 to 0.494) with the rs4939827 variant were associated with *SMAD7* expression enhancement regions. Liu Z et al. [16] showed greater binding of the H1-3 protein to the T allele of the rs12953717 variant, which is associated with lower *SMAD7* expression. On the other hand, they showed that the rs8085824 variant (one of the four in the work by Fortini BK et al.) in LD (r2 > 0.8) with the rs12953717 variant increases affinity for the heterogeneous nuclear ribonucleoprotein K (HNRNPK) with increased *SMAD7* expression. This variant was associated with an increased risk of CRC in univariate analysis in the work by Alidoust M et al. [17], but, when controlled for other risk factors, it lost this association, and when they studied the haplotype effect together with the rs8088297 and rs4939827 variants, both the T and C alleles were associated with increased risk depending on the alleles present in the other SNPs.

Our study did not replicate the association reported in two meta-analyses of these variants with the risk of CRC. This could be due to the small population size, genetic background differences in the population, or other lifestyle and environmental risk factors. As far as the prognostic effect is concerned, the evidence is limited, and one of the main limitations of our study was the fact that we did not have a sufficient sample size to control the results for disease stage and other variants.

## Conclusions

This study shows an association between both the CT genotype of the rs4939827 variant and the T allele of the rs12953717 variant and an increased risk of CRC. No association between *SMAD*7 variants and patient outcomes was observed.

More studies are needed to understand the effect of *SMAD7* variants on CRC risk in the Portuguese population and to corroborate their prognostic effect in the disease course.
